# Multiple Fractures in an Infant With Hepatoblastoma and Beckwith-Wiedemann Syndrome

**DOI:** 10.1210/jcemcr/luad110

**Published:** 2023-09-27

**Authors:** Toni Eimicke, Jonathan Swartz

**Affiliations:** Pediatric Endocrinology, The Barbara Bush Children's Hospital at Maine Medical Center, Portland, ME 04102, USA; Pediatric Endocrinology and Diabetes, The Barbara Bush Children's Hospital at Maine Medical Center, Portland, ME 04102, USA

**Keywords:** hepatoblastoma, fracture, prematurity, bone health in children

## Abstract

Children with hepatoblastoma have an increased incidence of fractures, but data are limited. Previous reports document an average of 4 fractures per child with hepatoblastoma. We present a severe case of a premature 4-month-old with multiple fractures in the setting of Beckwith-Wiedemann syndrome and hepatoblastoma. Although prematurity is a known risk for metabolic bone disease, it did not entirely explain the severity. Our patient underwent chemotherapy and surgical resection of his hepatoblastoma. Once deemed stable, he received a dose of zoledronic acid (ZA). One month post treatment with ZA, a skeletal survey revealed healing of the rib and femoral fractures and no new fractures. Five months post ZA, the skeletal survey revealed no new fractures and motor development was appropriate. An extensive search revealed scant literature on the rate or cause of pathologic fractures in patients with newly diagnosed hepatoblastoma. A better understanding of fracture risk in this population may guide prevention strategies, screening, and treatment. In our case, prematurity and substantial chronic illness may have compounded the known fracture risk associated with hepatoblastoma and may provide insight into the pathophysiology and prevention of fractures in this setting.

## Introduction

Reports in the literature are limited on the occurrence of fractures in children with hepatoblastoma, though an increased incidence of fractures has been previously reported. A retrospective study published in 2018 concluded that unexpected fractures in newly diagnosed patients with hepatoblastoma were relatively common, with a reported fracture rate of 17.8% [1]. Our aim is to add to the current literature to help better understand this phenomenon.

## Case Presentation

Our pediatric endocrinology team was initially asked to consult on the case of a 4-month-old infant with hypoglycemia in the setting of presumed (clinical diagnosis) Beckwith-Wiedemann syndrome (BWS) with recently diagnosed hepatoblastoma. Though it was not the primary reason for referral, our patient was also noted to have a history of a vertebral compression fracture on radiograph at T5 with 50% loss of vertebral body height. This initial fracture was noted at age 4 months on radiograph at the time of his hepatoblastoma diagnosis. On further investigation soon after diagnosis, our patient was noted to have multiple vertebral fractures. Approximately 1 month after diagnosis, he was noted to have anteromedial bilateral C6 through C8 rib fractures. Five months post diagnoses he was noted to have new bilateral proximal metaphyseal femur fractures. He was ultimately found to have diffuse osteopenia noted on imaging that included radiograph, nuclear medicine bone whole-body imaging, and bone survey. In all, his fractures included bilateral sixth through eighth rib fractures, compression fractures at T5, T10, and L5 [3], and bilateral proximal metaphyseal fractures at the proximal femurs [2]. Metastatic disease was a concern in the setting of hepatoblastoma, but imaging was not consistent with this.

Our patient was born premature at 24 5/7 weeks’ twin gestation with a birth weight of 860 g, which is classified as very low birth weight. His medical history was also notable for chronic lung disease of prematurity, patent foramen ovale, and features seen in BWS including macroglossia and right hemihypertrophy.

## Diagnostic Assessment

A clinical diagnosis of BWS was made following initial negative genetic testing. Methylation studies and CDK N1C molecular analysis were normal, indicating likely somatic mosaicism, thus further workup was pursued and a microarray was completed and was normal. Methylation studies and CDK N1C molecular analysis were repeated and sent for special analysis and were also negative. As our patient's blood and buccal testing was negative, affected liver (tumor and enlarged liver) tissue was tested and revealed gain of methylation at IC1 and loss of methylation at IC2 and a microarray obtained on liver tissue identified a mosaic loss of heterozygosity at 11p15.5p11.2. This all supported his diagnosis of BWS caused by mosaic, segmental paternal uniparental isodisomy of chromosome 11p.15.5, also called upd(11)pat.

Our patient was noted to have an enlarged and firm abdomen, which prompted imaging that revealed a “massively enlarged, heterogeneous liver highly concerning for large mass lesion such as hepatoblastoma.” The pediatric oncology team was consulted and a liver biopsy confirmed this diagnosis. He was started on treatment protocol AHEP1531, which consisted of cisplatin monotherapy. He also received a short course of dexamethasone that consisted of 1 mg dexamethasone every 12 hours for a total of 6 doses. While we cannot exclude the dexamethasone as a contributing factor, we did not consider the exposure to be significant enough to result in this degree of bone fragility and fractures. Repeat magnetic resonance imaging of the abdomen revealed minimal response to his initial treatment with cisplatin, thus his regimen was intensified. Doxorubicin, fluorouracil, and vincristine were added to the cisplatin and there was resultant shrinking of his tumor. Surgical resection followed at around age 8 months.

Our patient had transient hyperinsulinemic hypoglycemia for which he did not require any pharmacological intervention. He received gastrostomy-tube feedings primarily during infancy and progressed to oral feedings gradually. He completed a fast prior to discharge. He was confirmed to be adrenally sufficient with low-dose adrenocorticotropin stimulation testing.

Our patient's bone health laboratory values were unrevealing (Table 1). Initially his parathyroid hormone was elevated, but normalized on repeat evaluation without intervention. The alkaline phosphatase level was low normal for age. The magnesium was slightly low, but not significantly. The spot urine calcium/creatinine ratio was 0.41, appropriate for age. His weight for length remained above the 50th percentile and often near the 75th percentile throughout the hepatoblastoma treatment duration. Gestation-adjusted length for age improved from a *Z* score of −2.4 at the start of treatment to −1.4 at the conclusion of treatment.

**Table 1.** Bone health laboratory evaluation

Lab test—units (reference range)	Initial evaluation, age 4 months	1 wk later	1 mo later, age 5 months
Calcium—mg/dL (mmol/L) (8.8-10.8 mg/dL; 2.2-2.7 mmol/L)	10.2 (2.5)	9.9 (2.47)	9.3 (2.32)
Magnesium—mg/dL (mmol/L) (1.6-2.8 mg/dL; 0.66-1.2 mmol/L)	1.3 (0.53)		1.7 (0.7)
Parathyroid hormone intact—pg/mL (pmol/L) (15-65 pg/mL; 1.6-6.9 pmol/L)	116.9 (12.4)	68.3 (7.2)	58.1 (6.16)
Alkaline phosphatase—U/L (117-390 U/L)	145		149
25-Hydroxyvitamin D3—ng/mL (nmol/L)			35 (87.4)

## Treatment

Our patient received zoledronic acid (ZA) approximately 4 months after initial identification of fractures. Treatment of his fractures was repeatedly delayed due to complications of his oncology treatment and his unstable state. Once he was deemed stable enough for bisphosphonate treatment, he received one course of ZA at a dose of 0.0125 mg/kg, which he tolerated well [2, 3].

## Outcome and Follow-up

A skeletal survey obtained approximately 1 month after the dose of ZA revealed healing of the rib and femoral fractures and did not identify any new fractures. A subsequent skeletal survey obtained 5 months post treatment again revealed no new fractures, and our patient was noted to have normal motor developmental progress. Now 3 years post treatment, our patient continues to thrive without evidence of new fractures and with normal linear growth velocity. He did have a recurrence of his hepatoblastoma, but no associated bone health complications were noted.

## Discussion

While the finding of fractures in the setting of hepatoblastoma has been reported, we present a case of severe fractures unlike previously reported cases. In our patient, prematurity and a short course of glucocorticoid treatment posed additional risk factors for osteopenia, which we hypothesize contributed to the severity of his bone fragility. This degree of severity and the pattern of fractures is not commonly seen in infants with prematurity alone. Our patient had a total of 11 radiographically confirmed fractures. This is more than what we found to be noted in the literature for individual patients. One study revealed a rib fracture incidence of 0.3% [4] in very low birth weight premature infants. Another study noted a fracture rate of 0.6% [5] with 10 out of 1698 infants affected. Of those 10 infants in this study, 9 suffered multiple fractures. Still, of those 9 with multiple fractures, the total among all infants with fractures was 37, hence an average of approximately 4 fractures each. Lastly, Towbin et al [1] reported an average of 4.9 fractures per patient in their population of children with newly diagnosed hepatoblastoma. The range of fractures overall was 1 to 13. It is worth noting that 17.8% of the patients sustained fractures within 50 days prior to hepatoblastoma diagnosis or up to 2 weeks after diagnosis. The authors of the study noted there was no correlation between presence of fractures and age, sex, specific laboratory values, or tumor characteristics [1]. All fractures were identified at the baseline time point before initiation of therapy for hepatoblastoma, whereas our patient continued to experience new fracture onset throughout treatment [4].

Other risk factors present in our patient included chronic illness as well as immobility. Oral glucocorticoid exposure was not a major risk factor as he received only 2 short courses of dexamethasone, though this cannot be ignored or excluded as a contributing factor.

It is important that pediatric oncology providers who treat patients with hepatoblastoma be aware of the increased risk of fractures in this setting; however, the health care teams for this group of patients extend beyond the pediatric oncology team and those other team members must also be aware of this connection. In response to the severity of this case and the importance of bone health management, our institution convened a neonatal intensive care unit (NICU) bone health team. The goal of the bone health team is to better identify, monitor, and address bone health concerns in at-risk infants. Our bone health team is composed of NICU providers, pediatric endocrinology providers, registered dietitians, and pediatric gastroenterology providers.

## Learning Points

- Our case highlights the importance of bone health evaluations in at-risk patient populations, including those infants with newly diagnosed hepatoblastoma.
- Medical teams should consider having a lower threshold to evaluate hepatoblastoma patients for bone health concerns.
- The addition of a bone health team in the NICU setting can help increase awareness and identify patients at risk for poor bone health.

## Contributors

All authors made individual contributions to authorship. T.E. and J.S. were involved in the diagnosis and management of the patient. All authors reviewed and approved the final draft.

## Data Availability

Data sharing is not applicable to this article as no datasets were generated or analyzed during the current study.

## Abbreviations

BWS: Beckwith-Wiedemann syndrome
NICU: neonatal intensive care unit
ZA: zoledronic acid
